# Clinical decision-making in patients with non-ST-segment-elevation myocardial infarction: more than risk stratification

**DOI:** 10.3389/fcvm.2024.1382374

**Published:** 2024-10-23

**Authors:** Guangze Xiang, Gaoyang Cao, Menghan Gao, Tianli Hu, Wujian He, Chunxia Gu, Xulin Hong

**Affiliations:** ^1^ Department of Cardiology, Heart Center, First Affiliated Hospital of Wenzhou Medical University, Wenzhou, China; ^2^ Department of Colorectal Surgery, Sir Run Run Shaw Hospital, School of Medicine, Zhejiang University, Hangzhou, China; ^3^ Department of Cardiology, Sir Run Run Shaw Hospital, School of Medicine, Zhejiang University, Hangzhou, China; ^4^ Department of Cardiology, Hangzhou Red Cross Hospital, Hangzhou, China

**Keywords:** ACS—ACS/NSTEMI, total occlusion, percutaneous coronary intervention (PCI), multivessel disease (MVD), regional wall motion abnormalities (RWMAs)

## Abstract

**Objective:**

This study aims to explore the association between risk stratification and total occlusion (TO) of the culprit artery and multivessel disease (MVD) in patients with non-ST-segment-elevation myocardial infarction (NSTEMI) and to obtain more data on clinical decision-making in addition to risk stratification.

**Methods:**

We retrospectively collected data from 835 patients with NSTEMI admitted to our hospital between 1 January 2016 and 1 August 2022. All patients underwent percutaneous coronary intervention (PCI) within 72 h of admission. We excluded patients with a history of cardiac arrest, myocardial infarction, coronary artery bypass grafting, or PCI. Univariate and multivariate regression analyses were performed to determine the predictors of acute TO and MVD.

**Results:**

A total of 349 (41.8%) patients presented with a TO culprit vessel, whereas 486 (58.2%) had a patent culprit vessel. Thrombolysis in myocardial infarction (TIMI) and Global Registry of Acute Coronary Events (GRACE) risk stratifications were similar between the two groups of patients (*P* = 0.712 and 0.991, respectively). The TO infarct vessel was more commonly observed in the left circumflex artery. Patients with TO were more likely to develop MVD (*P* = 0.004). Univariate and multivariate linear regression analyses were performed to evaluate the role of variables in the presence of TO and MVD in patients with NSTEMI. Regional wall motion abnormalities (RWMAs) [odds ratio (OR) = 4.022; confidence interval (CI): 2.782–5.813; *P* < 0.001] were significantly linked to TO after adjusting for potentially related variables. Furthermore, age (OR = 1.032; CI: 1.018–1.047; *P* < 0.001), hypertension (OR = 1.499; CI: 1.048–2.144; *P* = 0.027), and diabetes mellitus (OR = 3.007; CI: 1.764–5.125; *P* < 0.001) were independent predictors of MVD in patients with NSTEMI. TIMI and GRACE risk scores were related to MVD prevalence in the multivariate logistic regression model. Patients with a TO culprit vessel had a higher risk of out-of-hospital cardiac death after a 2-year follow-up compared with those without a TO culprit vessel (*P* = 0.022).

**Conclusion:**

TIMI and GRACE risk scores were not associated with a TO of the culprit artery; however, they correlated with the prevalence of MVD in patients with NSTEMI. RWMA is an independent predictor of acute TO in patients with NSTEMI. Patients with a TO culprit vessel had worse clinical outcomes than those without a TO culprit vessel.

## Introduction

Acute coronary syndrome (ACS) is a prevalent cause of mortality worldwide (1). ACS is categorized into ST-segment-elevation myocardial infarction (STEMI), non-ST-segment-elevation myocardial infarction (NSTEMI), and unstable angina (UA) based on electrocardiogram (ECG) findings and cardiac markers. ST-segment elevation in two or more contiguous leads denotes total or near-total occlusion (TO) of the culprit artery, an indication for immediate coronary angiography and revascularization (2). Emergency intervention is recommended only for high-risk patients with NSTEMI, as assessed by the thrombolysis in myocardial infarction (TIMI) or Global Registry of Acute Coronary Events (GRACE) risk score (3). The reason for such a recommendation for this patient category is that the absence of ST-segment elevation is interpreted as a lack of TO. However, in clinical practice, coronary angiography reveals that up to 30% of patients with NSTEMI have total coronary occlusion (4, 5). Growing evidence suggests that NSTEMI patients with TO have a higher risk of mortality and major adverse cardiac events (6). However, only a limited number of dependable markers or methods are available to predict the occurrence of TO in individuals with NSTEMI. This delays the provision of reperfusion therapy for many patients. In addition, multivessel disease (MVD) in patients with NSTEMI also confers a poor prognosis on them (7, 8). Furthermore, information about the predictors of MVD in patients with NSTEMI is limited. Therefore, this study aims to explore the association of risk stratification with TO and MVD and to find more data on clinical decision-making in patients with NSTEMI in addition to risk stratification.

## Methods

### Study design

This retrospective study was conducted on consecutive patients with NSTEMI who were admitted to our emergency room between 1 January 2016 and 1 August 2022. The inclusion criteria were as follows: established diagnosis of NSTEMI and a need for interventional treatment within 72 h of admission, as recommended by the relevant guidelines. Exclusion criteria were as follows: previous myocardial infarction, coronary artery bypass grafting, or percutaneous coronary intervention (PCI). This study was approved by the Institutional Ethics Committee of the Sir Run Run Shaw Hospital. Informed consent was obtained from all patients enrolled in this study.

### Data collection and definitions

The demographic features of all patients, such as age, gender, body mass index (BMI), history of hypertension or diabetes, smoking, and family history of coronary artery disease (CAD), were obtained from electronic medical records. Blood samples were drawn from the patients upon their arrival at the emergency department via a peripheral venous line and processed immediately. ECG data on ST-segment depression, flat T waves or T-wave inversion, and pathological Q wave and echocardiographic data consisting of ejection fraction (EF), regional wall motion abnormalities (RWMAs), and left ventricular end-diastolic dimension (LVEDD) were extracted. Moreover, the TIMI (http://www.timi.org/index.php?page=calculators) and GRACE scores (https://www.outcomes-umassmed.org/grace/acs_risk/) were calculated separately for all patients.

Two experienced interventional cardiologists identified the culprit vessel using coronary angiography, ECG, and echocardiogram. TO was defined as a culprit vessel with a TIMI flow of 0–1, indicating no dye penetration or minimal dye penetration without complete vessel opacification. MVD was defined as significant stenosis (>70%) in two or more major coronary arteries of 2.5 mm diameter or more. NSTEMI was defined as elevated troponin levels and the absence of ST elevation at the time of diagnosis according to 2014 NSTEMI guidelines (3). According to the guidelines of the American Society of Echocardiography (9), longitudinal shortening or radial thickening of the myocardium after aortic valve closure (postsystolic shortening or thickening, sometimes referred to as tardokinesis) of >20% of the total deformation during the cardiac cycle is a consistent sign of regional functional inhomogeneity. And also, RWMAs were defined as the occurrence of hypokinetic (reduced thickening), akinetic (absent or negligible thickening), and dyskinetic (systolic thinning or stretching) which were detected by experienced sonographers and confirmed by trained cardiologists. According to the severity of RWMAs, we defined the RWMA score as normal = 1, hypokinetic = 2, akinetic = 3, and dyskinetic = 4.

### Follow-up and study endpoint

Detailed in-hospital and follow-up data were recorded and entered into a database. Patients were followed up through clinical visits or telephone calls to the referring physician. The clinical endpoint was cardiac death, which included death due to myocardial infarction, ventricular tachycardia/ventricular fibrillation, sudden cardiac arrest, or heart failure.

### Statistical analysis

Categorical variables were expressed as percentages and compared using the chi-square test. Continuous variables were expressed as a mean ± SD or median with interquartile ranges and were compared using Student's *t*-test or the Wilcoxon rank-sum test as appropriate. The independent predictors of acute TO and MVD were confirmed by using the multivariate logistic regression model after adjusting for the potentially related variables (*P* < 0.2 and *P* < 0.05, respectively) identified in the univariate analysis. Kaplan–Meier survival curves were compared using the log-rank test. SPSS V.26.0 was used for all analyses, and a value of *P* < 0.05 was considered statistically significant.

## Results

### Baseline and angiographic characteristics

**Figure 1:**
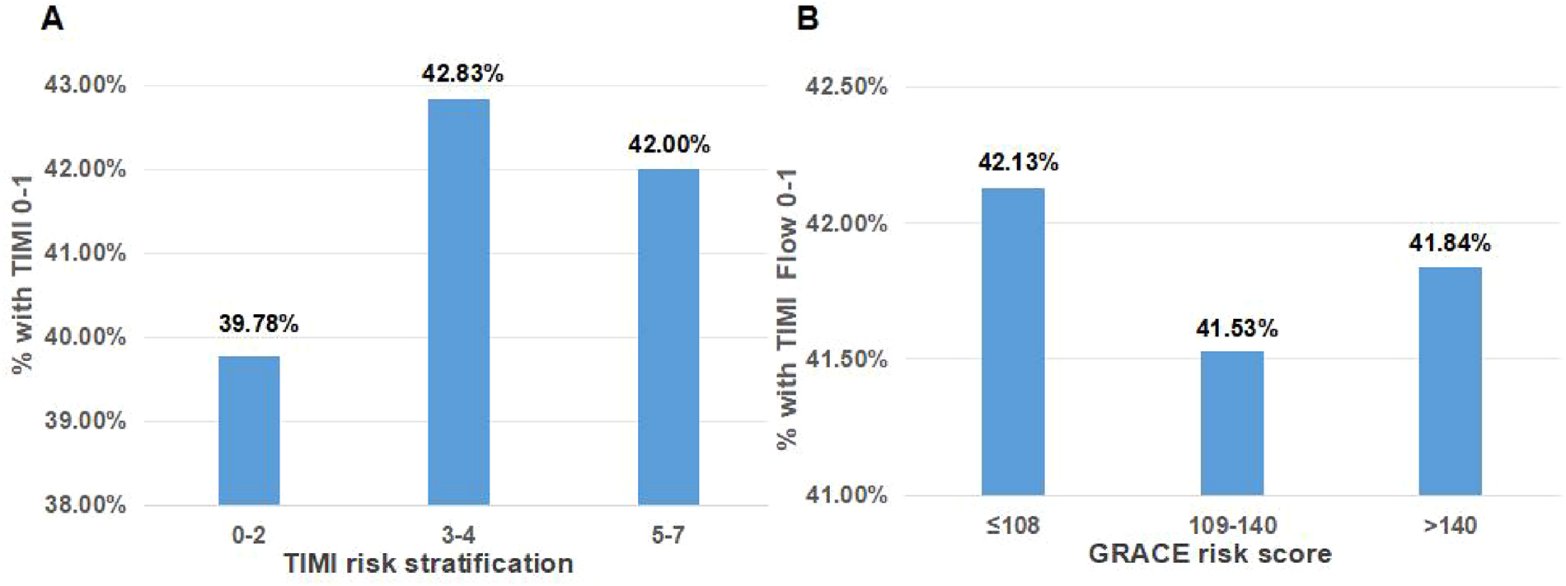
The proportion of patients with an occluded culprit artery by TIMI risk stratification **(A)** and the GRACE risk score **(B)**.

The study population comprised 835 patients with NSTEMI: 349 (41.8%) presented with a TO culprit vessel and 486 (58.2%) had a patent culprit vessel. Baseline characteristics according to the patency of the culprit artery are listed in Table 1. The two groups showed similarities with respect to demographic characteristics and clinical data, and there were no significant differences between them, although differences were noticed with regard to BMI, white blood cell (WBC) count, neutrophil count, low-density lipoprotein cholesterol (LDL-C) level, high-sensitivity C-reactive protein (hs-CRP) level, heart rate (HR), and RWMAs, which were all higher or more commonly detected in patients with TO. TIMI and GRACE risk stratification were similar in both groups (*P* = 0.712 and 0.991, respectively). The proportion of patients with a TO of the culprit artery divided by different types of risk stratification is shown in Figures 1A,B.

**Table 1:** Baseline and angiographic characteristics.

Variable	Occluded culprit Coronary artery		*P* value
	Yes	No	
	TIMI flow 0–1 (*n* = 349)	TIMI flow 2–3 (*n* = 486)	
Demographic characteristics			
Age (years)	62.4 ± 13.3	63.5 ± 12.8	0.209
Male	281 (80.5%)	364 (74.9%)	0.056
BMI	24.8 ± 3.9	24.2 ± 3.9	0.033
Hypertension	216 (61.9%)	307 (63.2%)	0.707
Diabetes mellitus	79 (22.6%)	114 (23.5%)	0.781
Hyperlipidemia	79 (22.6%)	88 (18.1%)	0.107
Current smoker	165 (47.3%)	198 (40.7%)	0.06
Family history of CAD	6 (1.7%)	12 (2.5%)	0.462
Laboratory testing			
WBC, 10^9^/L	9.2 (7.2–11.3)	8.1 (6.5–9.9)	<0.001
Neutrophil (%)	74.7 ± 9.9	72.2 ± 10.5	0.001
PLT, 10^9^/L	199 (170–241)	198 (157–240)	0.215
LDL-C, mmol/L	2.60 (2.03–3.33)	2.50 (1.92–3.06)	0.016
VLDL-C, mmol/L	0.57 (0.34–0.77)	0.53 (0.29–0.85)	0.641
HDL-C, mmol/L	0.94 (0.81–1.11)	0.96 (0.81–1.13)	0.681
Fibrinogen, g/L	3.48 (2.99–4.31)	3.45 (2.91–4.02)	0.104
DDi, μmol/L	0.33 (0.20–0.57)	0.36 (0.23–0.67)	0.089
NT-proBNP, pg/ml	825 (292–2,019)	660 (197–1,883)	0.073
hs-CRP, mg/L	3.4 (1.4–11.7)	2.6 (1.0–9.3)	0.012
Troponin I, ug/L	0.76 (0.19–3.12)	0.63 (0.16–2.79)	0.082
Clinical status on admission			
Killip class			0.325
I	271 (77.7%)	358 (73.7%)	
II	49 (14.0%)	81 (16.7%)	
III	14 (4.0%)	30 (6.2%)	
IV	15 (4.3%)	17 (3.5%)	
MAP, mmhg	92 ± 15	93 ± 14	0.423
HR, /min	80 ± 17	78 ± 15	0.027
ECG changes			
ST depression ≥ 1 mm	110 (31.5%)	130 (26.7%)	0.133
Flat T waves or T-wave inversion	121 (34.7%)	170 (35.0%)	0.926
Pathological Q wave	48 (13.8%)	60 (12.3%)	0.55
Echocardiographic data			
RWMAs	205 (58.7%)	108 (22.2%)	<0.001
RWMA score	1.79 ± 0.76	1.23 ± 0.49	<0.001
Left ventricular enlargement	37 (10.6%)	49 (10.1%)	0.808
EF (%)	60.6 ± 10.4	61.2 ± 10.4	0.394
TIMI risk score			0.712
0–2	107 (30.7%)	162 (33.3%)	
3–4	221 (63.3%)	295 (60.7%)	
5–7	21 (6.0%)	29 (6.0%)	
GRACE risk score			0.991
≤108	83 (23.8%)	114 (23.5%)	
109–140	125 (35.8%)	176 (36.2%)	
>140	141 (40.4%)	196 (40.3%)	
Culprit coronary artery			<0.001
LAD	108 (30.9%)	297 (61.1%)	
LCX	131 (37.5%)	83 (17.1%)	
RCA	110 (31.5%)	106 (21.8%)	
Number of diseased vessels			0.010
1	71 (20.3%)	141 (29.0%)	
2	99 (28.4%)	137 (28.2%)	
3	179 (51.3%)	208 (42.8%)	
Lesion morphology			
Thrombus	112 (32.1%)	51 (10.5%)	<0.001
Calcium	31 (8.9%)	73 (15.0%)	0.008
Collaterals present	74 (21.2%)	38 (7.8%)	<0.001
Glycoprotein IIb/IIIa inhibitor use	113 (32.4%)	44 (9.1%)	<0.001
Angiography and revascularization time, hour	35.4 ± 6.7	36.6 ± 7.2	0.302

BMI, body mass index; CAD, coronary artery disease; WBC, white blood cell; PLT, platelet; LDL-C, low-density lipoprotein cholesterol; VLDL-C, very low-density lipoprotein cholesterol; HDL-C, high-density lipoprotein cholesterol; DDi, d-dimer; hs-CRP, high-sensitivity C-reactive protein; MAP, mean arterial pressure; HR, heart rate; ECG, electrocardiogram; RWMAs, regional wall motion abnormalities; EF, ejection fraction; LAD, left atrial diameter; LCX, left circumflex; RCA, right coronary artery.

The angiographic characteristics of the patient groups are presented in Table 1. TO was more commonly observed in the left circumflex artery (LCX). Patients with a TO of the culprit artery had a higher incidence of MVD (*P* = 0.004), a greater number of thrombotic culprit lesions (*P* < 0.001), high levels of collateral circulation (*P* < 0.001), and fewer calcified lesions (*P* = 0.008). In the TO group, glycoprotein IIb/IIIa inhibitors were used more frequently.

### Risk factors associated with TO and MVD and predictors of MVD

Univariate and multivariate linear regression analyses were performed to evaluate the role of variables in NSTEMI patients with a TO of the culprit artery. RWMA [odds ratio (OR) = 4.022, confidence interval (CI): 2.782–5.813; *P* < 0.001] was significantly associated with TO after adjusting for potentially related variables (Table 2). We also examined the variables through univariate and multivariate regression analyses and found that age (OR = 1.032, CI: 1.018–1.047; *P* < 0.001), hypertension (OR = 1.499, CI: 1.048–2.144; *P* = 0.027), and diabetes mellitus (OR = 3.007, CI: 1.764–5.125; *P* < 0.001) were independent predictors of MVD in patients with NSTEMI (Table 3). An assessment of the predictive value of MVD by risk stratification revealed that the TIMI and GRACE risk scores were associated with the prevalence of MVD in the multivariate logistic regression model (Table 4).

**Table 2:** The risk factors associated with TO in patients with NSTEMI.

Variables	Univariable		Multivariable	
	OR (95% CI)	*P*-value	OR (95% CI)	*P*-value
Male	1.385 (0.991–1.936)	0.057	1.125 (0.677–1.868)	0.649
BMI	1.043 (1.003–1.084)	0.035	1.035 (0.985–1.028)	0.558
Current smoker	1.304 (0.988–1.721)	0.06	1.230 (0.816–1.854)	0.322
WBC	1.096 (1.049–1.145)	<0.001	1.076 (0.994–1.165)	0.072
Neutrophil	1.025 (1.010–1.040)	0.001	1.006 (0.985–1.028)	0.558
PLT	1.002 (1.000–1.004)	0.116	0.999 (0.996–1.002)	0.529
LDL-C	1.173 (1.024–1.344)	0.021	1.088 (0.907–1.305)	0.336
Fibrinogen	1.159 (1.020–1.318)	0.024	1.083 (0.914–1.284)	0.358
HR	1.010 (1.001–1.018)	0.028	1.003 (0.991–1.015)	0.639
ST depression ≥ 1 mm	1.260 (0.932–1.705)	0.133	0.735 (0.474–1.140)	0.169
RWMAs	4.983 (3.684–6.739)	<0.001	4.069 (2.719–6.088)	<0.001

TO, total occlusion; NSTEMI, non-ST-segment-elevation myocardial infarction; BMI, body mass index; WBC, white blood cell; PLT, platelet; LDL-C, low-density lipoprotein cholesterol; HR, heart rate; RWMAs, regional wall motion abnormalities.

**Table 3:** The risk factors associated with MVD in patients with NSTEMI.

Variables	Univariable		Multivariable	
	OR (95% CI)	*P*-value	OR (95% CI)	*P*-value
Age (years)	1.041 (1.028–1.054)	<0.001	1.032 (1.018–1.047)	<0.001
Hypertension	1.972 (1.437–2.707)	<0.001	1.499 (1.048–2.144)	0.027
Diabetes mellitus	3.936 (2.381–6.508)	<0.001	3.007 (1.764–5.125)	<0.001
Fibrinogen	1.334 (1.125–1.582)	0.001	1.105 (0.888–1.374)	0.371
hs-CRP	1.011 (1.003–1.019)	0.009	1.003 (0.993–1.013)	0.55
Killip class (>1)	1.691 (1.141–2.505)	0.009	1.224 (0.784–1.911)	0.374
Left ventricular enlargement	1.848 (1.019–3.351)	0.043	1.542 (0.767–3.104)	0.224

MVD, multivessel disease; NSTEMI, non-ST-segment-elevation myocardial infarction; hs-CRP, high-sensitivity C-reactive protein.

**Table 4:** Predictors of MVD by TIMI and GRACE risk stratification.

Variables	Univariable		Multivariable	
	OR (95% CI)	*P*-value	OR (95% CI)	*P*-value
TIMI risk stratification				
0–2	Reference	—		
3–4	2.372 (1.713–3.283)	<0.001	2.262 (1.594–3.209)	<0.001^a^
5–7	14.892 (3.543–62.582)	<0.001	12.160 (2.868–51.552)	0.001^a^
GRACE risk score				
≤108	Reference	—		
109–140	1.867 (1.269–2.746)	0.002	1.783 (1.165–2.728)	0.008^b^
>140	3.087 (2.064–4.616)	<0.001	2.433 (1.564–3.786)	<0.001^b^

MVD, multivessel disease; hs-CRP, high-sensitivity C-reactive protein.

^a^ Adjusted for fibrinogen, hs-CRP, left ventricular enlargement.

^b^ Adjusted for hypertension, diabetes mellitus, fibrinogen, hs-CRP, left ventricular enlargement.

### Outcomes in the TO and non-TO groups of patients

**Figure 2:**
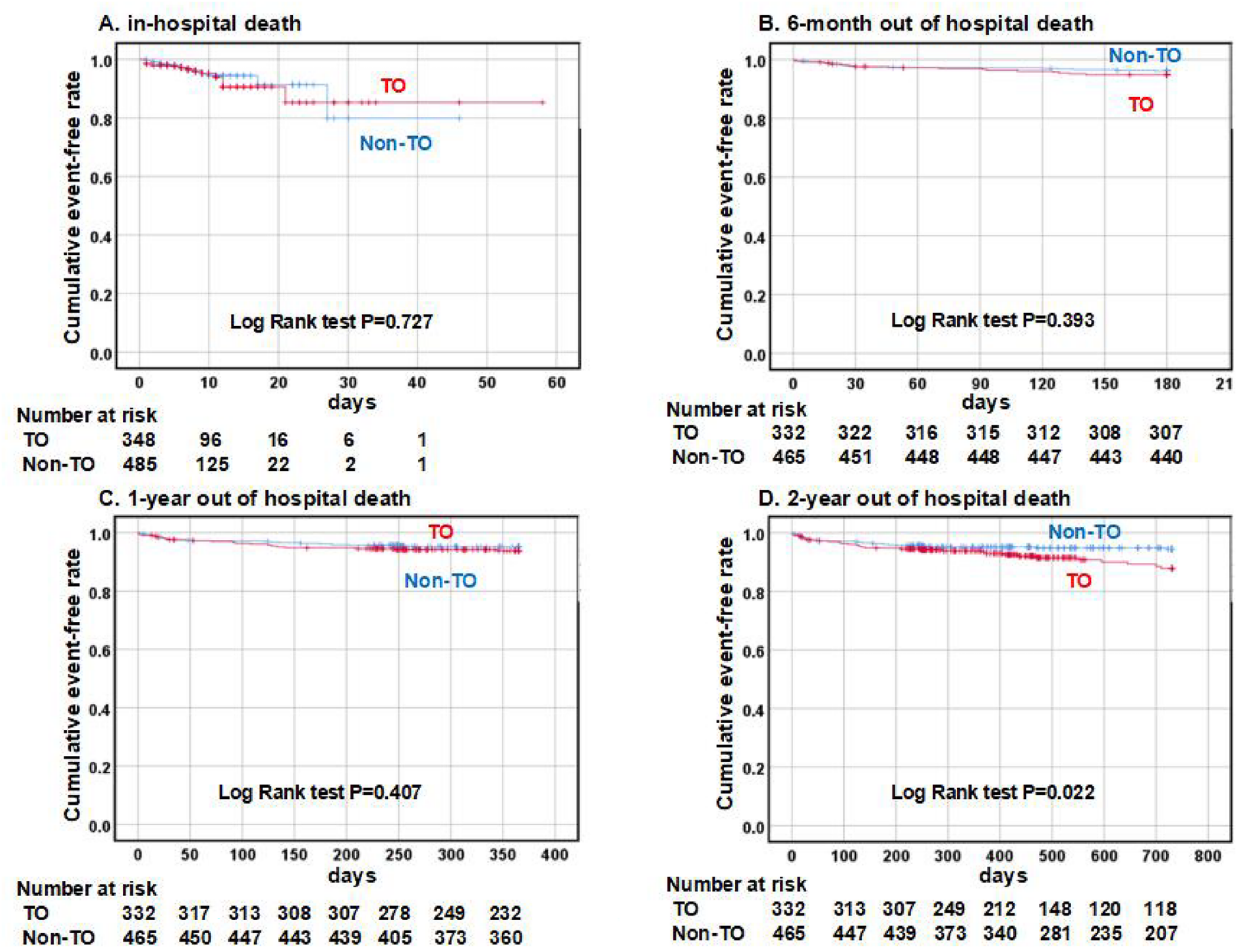
A comparison of cardiac death between patients belonging to the TO and the non-TO groups. A Kaplan–Meier analysis of cardiac death demonstrated similar outcomes in the TO group (red line) and the non-TO group (blue line) in terms of in-hospital, 6-month, and 1-year follow-ups **(A–C)**. The TO group had a higher rate of cardiac death after a 2-year follow-up compared with the non-TO group **(D)**. TO indicates total occlusion of the culprit artery.

The outcomes of the 2-year follow-up period were compared between the TO and the non-TO groups of patients. A Kaplan–Meier analysis showed similar rates of in-hospital, 6-month out-of-hospital, and 1-year out-of-hospital cardiac deaths within the two groups (*P* = 0.727, 0.393, and 0.407, Figures 2A–C). However, patients with a TO culprit vessel had a higher rate of out-of-hospital cardiac death after the 2-year follow-up compared with those without a TO culprit vessel (*P* = 0.022, Figure 2D).

## Discussion

### Main findings

The principal findings of this study are as follows: (1) the rate of prevalence of acute TO in patients with NSTEMI was 41.8%; (2) the LCX culprit vessel had the highest likelihood of TO in the setting of NSTEMI; (3) TIMI and GRACE risk stratification did not correlate with a TO of the culprit vessel but correlated with the prevalence of MVD; (4) RWMA was an independent predictor of TO in patients with NSTEMI; (5) patients with a TO of the culprit vessel had worse clinical outcomes at the mid-term follow-up compared with those without a TO of the culprit vessel.

### LCX is the vessel most likely to be occluded in NSTEMI

The reported rates of prevalence of acute TO in patients with NSTEMI ranged from 24.0% to 37.2% (4, 10–12). In our study, the frequency was higher than that reported in previous studies, which may be explained by the differences in patient selection and variation in the risk stratification of the patients. Our study found that the vessel most likely occluded in NSTEMI is the LCX, which is consistent with the results of previous trials (13, 14). This finding is perhaps attributed to the fact ischemia in the LCX territory is difficult to detect since 38%–47% of patients do not show ST-segment shifts in any standard ECG lead (15). At our center, we routinely obtained an 18-lead electrocardiogram for patients presenting to the emergency department with ACS. STEMI due to occlusion of the LCX was typically manifested on ECG as ST-segment elevations in lateral, inferior, and posterior leads, while NSTEMI due to occlusion of the LCX was defined as elevated troponin levels and the absence of ST-segment elevations in any standard ECG lead at the time of diagnosis.

### Analysis of the outcome for patients with TO

In patients with NSTEMI, a quantitative assessment of ischemic risk employing scores such as the GRACE risk score and TIMI risk stratification proved to be superior to clinical assessment alone. Current clinical practice guidelines for non-ST-segment-elevation acute coronary syndrome (NSTE-ACS) recommend different treatment strategies and timings for intervention according to the initial risk stratification (16). However, we demonstrated that the proportion of TO in different risk stratifications of TIMI and GRACE was similar, which accounted for the inability of the TIMI and GRACE risk scores to indicate whether the culprit vessel was totally occluded. This may result in delayed revascularization in non-high-risk patients. Prior studies have shown that the outcomes for NSTEMI patients with TO who do not receive timely revascularization are poorer than those for STEMI patients (17, 18). Moreover, a meta-analysis suggested that NSTEMI patients with totally occluded culprit vessels on coronary angiography were at a higher risk of mortality and major adverse cardiac events (19). Our findings were comparable but not identical to those of previous studies regarding the outcomes for patients with a TO culprit vessel. Patients with a TO culprit vessel had a similar rate of cardiac death in the hospital and at the short-term follow-up, while these patients had a higher risk of mortality in the mid-term follow-up compared with those without a TO culprit vessel. This could be attributed to the following: (1) all patients with NSTEMI received timely revascularization and proper medication in our center, contributing to a reduction in mortality after PCI and at the short-term follow-up. (2) Patients with a TO culprit vessel usually suffered a complete infarction of a part of the myocardium, requiring better treatment for myocardial remodeling. Some patients had a higher mortality rate due to poor drug adherence at the mid-term follow-up. Hence, finding reliable predictors of TO in patients with NSTEMI is of great significance.

### RWMA is an effective predictor to determine acute TO in NSTEMI

Echocardiography is a feasible tool for TO detection. Eek et al. (20) found that detection of contractile abnormalities by strain or conventional echocardiography might be a very useful method to identify acute TO in NSTEMI. In our study, RWMA was also shown to be an effective predictor of acute TO in patients with NSTEMI. The possible mechanism is that acute persistent coronary occlusion can cause more severe ischemia and impaired systolic function in large regions of the myocardium.

### TIMI and GRACE risk stratification correlated with MVD prevalence

The prognosis of NSTEMI in the presence of MVD is poor (3). Although risk factors for coronary heart disease are included in risk stratification, the association between the above-mentioned risk scores and coronary severity in NSTEMI has rarely been studied. A few studies have reported on the relationship between risk stratification and the extent of disease (21–25). Our study further evaluated the novel role of existing risk stratification. Identifying the predictors of severe NSTEMI is necessary for preoperative evaluation and developing postoperative preventive strategies based on risk factors.

### Study limitations

Our study had some limitations. First, this was a retrospective, single-center study; therefore, a larger sample size with cross-center comparisons is required to validate the results further. Second, the outcome of the patients stratified into two groups in our study was recorded only in terms of cardiac death; it was not recorded by also taking into account other major adverse cardiac and cerebrovascular events, such as recurrent angina, heart failure, and stroke. Detailed records of the causes of adverse events can help make better decisions.

## Conclusions

The TIMI and GRACE risk scores could not independently predict the TO of the culprit artery but were associated with the prevalence of MVD in patients with NSTEMI. RWMA was an independent predictor of acute TO in these patients. Patients with a TO culprit vessel had worse clinical outcomes at the mid-term follow-up compared with their non-TO counterparts.

## Data Availability

The raw data supporting the conclusions of this article will be made available by the authors without undue reservation.
